# Supplementary material for: Effect of Solvent Treatment on the Composition and Structure of Santanghu Long Flame Coal and Its Rapid Pyrolysis Products
Source: Molecules. 2023 Oct 13;28(20):7074. doi: 10.3390/molecules28207074 (PMC10609383; doi:10.3390/molecules28207074)
Supplement: Supplementary file 1 [file molecules-28-07074-s001.zip › molecules-2649208-supplementary.pdf]

Supplementary Material

Effect of Solvent Treatment on the Composition and Structure of Santanghu Long Flame Coal and Its Rapid Pyrolysis Products

Jia Guo [1,2], Meixia Zhu [2], Wenlong Mo [1,2,*], Yanxiong Wang [2], Junrong Yuan [2], Ronglan Wu [2], Junmin Niu [1,*], Kongjun Ma [2], Wencang Guo [1], Xianyong Wei [2,3], Xing Fan [2] and Naeem Akram [4]

[1] Xinjiang Energy Co., Ltd., Urumqi 830000, China; gj459824057@126.com (J.G.); hjgwc@126.com (W.G.)

[2] State Key Laboratory of Chemistry and Utilization of Carbon Based Energy Resources and Key Laboratory of Coal Clean Conversion & Chemical Engineering Process (Xinjiang Uyghur Autonomous Region), School of Chemical Engineering and Technology, Xinjiang University, Urumqi 830046, China; 18580890109@163.com (M.Z.); 18599119007@163.com (Y.W.); yuanjunrong321@163.com (J.Y.); wuronglan@163.com (R.W.); kjma@xju.edu.cn (K.M.); wei_xianyong@163.com (X.W.); fanxing@sdust.edu.cn (X.F.)

[3] Key Laboratory of Coal Processing and Efficient Utilization, Ministry of Education, China University of Mining & Technology, Xuzhou 221116, China

[4] School of Chemical Engineering, Minhaj University Lahore, Lahore 54000, Pakistan; naeemakram63@gmail.com

* Correspondence: mowenlong@xju.edu.cn (W.M.); njm555@139.com (J.N.)

Table S1

Alkanes detected in SLFC and SLFC-L

Species	Peak	Compound	CAS	RC (area %)	
				SLFC	SLFC-L
Normal alkanes (NAs)	**15**	Decane	124-18-5	2.06	1.90
	**23**	Undecane	1120-21-4	0.85	0.85
	**34**	Dodecane	112-40-3	1.25	1.09
	**40**	Tridecane	629-50-5	0.93	0.88
	**45**	Tetradecane	629-59-4	0.79	0.74
	**53**	Hexadecane	544-76-3	-	0.99
	**54**	Pentadecane	629-62-9	0.99	-
	**62**	Nonadecane	629-92-5	-	0.60
	**63**	Hexadecane	544-76-3	0.65	-
	**69**	Heptadecane	629-78-7	1.03	0.81
	**75**	Pentadecane	629-62-9	-	0.67
	**76**	Octadecane	593-45-3	0.77	-
	**79**	Eicosane	112-95-8	-	0.78
	**80**	Nonadecane	629-92-5	0.81	-
	**84**	Heneicosane	629-94-7	0.89	-
	**104**	Tetratetracontane	7098-22-8	6.76	5.64
Branched alkanes (BAs)	**49**	2,6,10-Trimethyldodecane	3891-98-3	-	0.56
	**50**	2,6,10-Trimethy ltridecane	3891-99-4	0.53	-
	**65**	2,6,10-Trimethyldodecane	3891-98-3	0.32	-
	**83**	3-Ethyl-5-(2-ethylbutyl)octade	55282-12-7	-	0.81
	**87**	Dodecane,5,8-diethyl-	24251-86-3	0.97	-
	**93**	Heptadecane,2,3-dimethyl-	61868-03-9	2.00	-
Cyclic alkanes	**33**	Nonylcyclopropane	74663-85-7	0.79	-
	**39**	1-Heptyl-2-methylcyclopropane	74663-91-5	0.89	-
	**106**	Cyclohexane,1,1'-dodecylidenebis[4-methyl-	55334-09-3	3.02	-
	**108**	Cyclohexane,1,3,5-trimethyl2-octadecyl-	55282-34-3	8.46	6.91

Table S2

Olefines detected in SLFC and SLFC-L

Peak	Compound	CAS	RC (area %)	
			SLFC	SLFC-L
**10**	2-Methylbicyclo[4.3.0]non-1(6)-ene	60223-07-6	0.26	-
**14**	1-Decene	872-05-9	0.57	-
**17**	p-Mentha-1,5,8-triene	21195-59-5	0.40	-
**18**	(3E,5E)-2,6-Dimethyl-1,3,5,7-octatetrene	460-01-5	-	0.88
**22**	3-Dodecene,(Z)-	7239-23-8	1.42	-
**32**	1-Dodecene	112-41-4	-	0.67
**38**	1-Tridecene	2437-56-1	-	0.85
**42**	Bicyclo[4.4.1]undeca-1,3,5,7,9-pentaene	2443-46-1	0.77	-
**44**	1-Tetradecene	1120-36-1	0.67	0.78
**52**	Cetene	629-73-2	0.57	0.60
**60**	1-Nonadecene	18435-45-5	-	0.42
**67**	1-Heptadecene	6765-39-5	-	0.42
**68**	5-Octadecene,(E)-	7206-21-5	0.36	-
**70**	5-Methyl-1-undecene	74630-38-9	-	2.26
**71**	Tridecane,7-methylene-	19780-80-4	2.35	-
**73**	Heneicosene	27400-79-9	-	0.53
**74**	1-Heptadecene	6765-39-5	0.45	-
**77**	1-Docosene	1599-67-3	-	0.63
**78**	1-Nonadecene	18435-45-5	0.55	-
**88**	17-Pentatriacontene	6971-40-0	-	0.70
**92**	1-Hexacosene	18835-33-1	-	1.09
**99**	17-Pentatriacontene	6971-40-0	2.13	-
**103**	Squalene	111-02-4	3.22	3.07

Table S3

Arenes detected in SLFC and SLFC-L

Species	Peak	Compound	CAS	RC (area %)	
				SLFC	SLFC-L
Monocyclic aromatics (MAs)	**1**	1,3,5-Cycloheptatriene	108-88-3	1.96	-
	**6**	p-Xylene	106-42-3	1.21	-
	**7**	1,2-xylene	95-47-6	-	1.55
	**8**	Ethylbenzene	100-41-4	1.74	-
	**11**	1-ethyl-2-methyl-Benzene	611-14-3	0.67	-
	**13**	Mesitylene	108-67-8	-	0.60
	**16**	Benzene,(1-methylethyl)-	98-82-8	0.77	-
	**20**	1,2,4,5-Tetramethylbenzene	95-93-2	0.36	-
	**26**	Benzene,2,4-diethyl-1-methyl-	1758-85-6	-	0.28
	**28**	Benzene, 1-ethyl-2,4-dimethyl-	874-41-9	0.81	-
	**51**	Benzene, octyl-	2189-60-8	-	0.63
	**56**	1,1,4,5,6-Pentamethyl-2,3-dihydro-1H-indene	16204-67-4	0.40	-
Polycyclic aromatic hydrocarbon (PAHs)	**30**	1H-Indene, 3-methyl-	767-60-2	0.75	1.23
	**41**	7H-Benzocycloheptene	264-09-5	-	0.81
	**43**	1-Methylnaphthalene	90-12-0	0.71	0.88
	**46**	Naphthalene,1-(2-propen-1-yl)-	2489-86-3	-	0.42
	**47**	1,4-Dimethylnaphthalene	571-58-4	0.34	-
	**48**	1,7-Dimethylnaphthalene	575-37-1	-	0.42
	**64**	1,4,5-Trimethylnaphthalene	2131-41-1	0.83	1.13
	**66**	Naphthalene,1,6-dimethyl-4-(1-methylethyl)-	483-78-3	0.28	-
	**90**	Retene	483-65-8	1.25	-

Table S4

Oxygen-containing organic compounds detected in SLFC and SLFC-L

Species	Peak	Compound	CAS	RC (area %)	
				SLFC	SLFC-L
Alcohols	**4**	Bicyclo[2.2.1]hept-2-en-7-ol	53783-87-2	0.26	-
	**5**	D-erythro-L-galacto-Octopyranose, a-(8CI)	5329-49-7	0.55	-
	**9**	Z,Z-2,5-Pentadecadien-1-ol	139185-79-8	0.49	-
	**21**	1-Undecanol	112-42-5	-	0.99
	**24**	3-Cyclohexen-1-ol,5-methylene-6-(1-methylethenyl)-	54274-41-8	0.34	-
	**31**	2-Hydroxytetralin	530-91-6	-	0.35
	**36**	(1-Benzyl-cyclopropyl)-methanol	108546-79-8	-	0.42
	**37**	1-Naphthalenol,1,2,3,4-tetrahydro-3-methyl-	3344-45-4	0.45	0.81
	**55**	2,9-Heptadecadiene-4,6-diyn-8-ol, (Z,E)-	50816-77-8	-	0.39
	**61**	Hexadecanol; 1-Cetanol	36653-82-4	0.38	-
	**72**	3,7,11-Trimethyl-1-dodecanol	6750-34-1	0.43	0.39
	**91**	Lycopene,1,2-dihydro-1-hydroxy	105-92-0	-	0.46
	**107**	1-Hentetracontanol	40710-42-7	-	2.61
	**109**	Octacosanol	557-61-9	-	26.60
	**110**	1-Hexacosanol	506-52-5	22.81	-
Carboxylic acids (CAs)	**27**	Methyl 4,6-tetradecadiynoate	NA	0.24	-
	**57**	2-Oxiraneoctanoic acid,3-octyl-, (2R,3S)-rel-	24560-98-3	-	0.28
	**58**	2,5-Octadecadiynoic acid,methyl ester	57156-91-9	-	0.39
	**59**	Naproxen	22204-53-1	0.42	-
	**81**	Ethyliso-allocholate	47676-48-2	0.47	-
	**82**	Kaur-16-ene-3,7,15,18-tetrol,7,18-diacetate, (3a,4b,7b,15a)- (9CI)	70902-37-3	-	0.46
	**85**	Ethyliso-allocholate	47676-48-2	-	1.27
	**86**	Lauric acid (1aR)-1aα,2,5,5a,6,9,10,10aα-octahydro-5aβ-hydroxy-4-hydroxymethyl-1,1,7,9α-tetramet	77508-68-0	-	0.85
	**95**	Oleic acid,eicosyl este	22393-88-0	-	1.48
	**96**	7,8-Epoxylanostan-11-ol,3-acetoxy-	NA	-	0.92
	**97**	Lauric acid (1aR)-1aα,2,5,5a,6,9,10,10aα-octahydro-5aβ-hydroxy-4-hydroxymethyl-1,1,7,9α-tetramet	77508-68-0	1.03	-
	**98**	9,19-Cyclolanostan-3-ol,24,24-epoxymethano-, acetate	NA	-	1.94
	**100**	5H-Cyclopropa(3,4)benz(1,2-e)azulen-5-one,1,1a-1b-4,4a,7a-7b,8,9,9a-decahydro-7b-9-9a-trihydroxy-3-hydroxymethyl-1,1,6,8-tetramethyl-4a-methoxy-,9,9a-	54870-24-5	-	2.47

		didecanoate			
	**101**	Oleic acid,eicosyl ester	22393-88-0	1.17	-
	**105**	Oleic acid,3-(octadecyloxy)propyl ester	17367-41-8	1.72	-
Ethers	**29**	Naphthalene,1,2,3,4-tetrahydro-1-methoxy-	1008-18-0	-	0.78
	**89**	5a-Cholestan-3-one, cyclic-1,2-ethanediylaetal	25328-53-4	0.81	-
	**94**	Tetradecane,1-(tetradecyloxy)-	5412-98-6	-	1.09
	**102**	1,1'-[Ethylenebis(oxy)]bis[(Z)-9-octadecene]	17367-13-4	6.48	-
Aldehydes	**25**	3-O-Benzyl-d-glucose	10230-17-8	0.59	-
	**35**	5,8-Decadien-2-one,5,9-dimethyl-,(E)-	130876 -99-2	0.43	-

Table S5

Other heteroatom-containing organic compounds detected in SLFC and SLFC-L

Peak	Compound	CAS	RC (area %)	
			SLFC	SLFC-L
**2**	1,1,2-trichloroethane	79-00-5	-	3.42
**3**	Tetrachloroethylene	127-18-4	-	5.92
**12**	P-methylampheamine	1964/11/9	0.40	-
**19**	Hexachloroethane	67-72-1	-	1.66
